# Extracellular space diffusion modelling identifies distinct functional advantages of glutamatergic and GABAergic synapse geometries

**DOI:** 10.1186/s12987-026-00797-3

**Published:** 2026-03-25

**Authors:** Paula Gimenez, Rohisha T. Shakya, Fidel Santamaria, Jan Tønnesen

**Affiliations:** 1 Neuronal Excitability Lab, Instituto Biofisika (CSIC-UPV), Leioa, 48940 Spain; 2 Aligning Science Across Parkinson’s (ASAP) Collaborative Research Network, Chevy Chase, MD 20815 USA; 3 Neuronal Excitability Lab, Achucarro Basque Center for Neuroscience, Leioa, 48940 Spain; 4 Department of Neuroscience, Faculty of Medicine and Nursing, University of the Basque Country (UPV/EHU), Leioa, 48940 Spain; 5 Department of Neuroscience, Developmental and Regenerative Biology, The University of Texas at San Antonio, San Antonio, TX 78349 USA

**Keywords:** Brain, Extracellular space, Molecular diffusion, Signaling, GABA, Glutamate

## Abstract

**Background:**

The brain extracellular space (ECS) is a convoluted compartment of nano- to microscale interconnected ducts filled with interstitial fluid and lined by neural cell membranes. A key step in signaling between neural cells is diffusion through the ECS of transmitter molecules released from point sources distributed throughout the parenchyma. Yet, signaling is generally considered solely from the stance of cellular properties, while disregarding ECS sojourn time and putative signal modulation at this phase. Where ECS diffusion is considered, it is commonly done based on volume-averaging techniques blind to individual signaling events or actual ECS structural geometry. This has precluded knowledge on how specific ECS geometries may impact diffusion and modulate signaling arising from individual transmitter release events. We hypothesized that ECS geometry can shape local diffusion gradients resulting from individual point source release events and thereby tune signaling, and we further propose that this modulation can impart non-random functionality.

**Methods:**

To access the scale of individual transmitter release events and true ECS geometries, we used super-resolution STED microscopy to image the ECS in the hippocampal CA1 *stratum radiatum* of live mouse brain slices. We then developed a computational diffusion model, *DifFlux*, based on super-resolved images of hippocampal ECS and applied this along single molecule Monte Carlo diffusion simulations. Our approach allows us to simulate diffusion of molecules of our choosing in actual live ECS geometries.

**Results:**

We observed local anomalous and anisotropic diffusion imposed by ECS geometry, whereby diffusion along larger structures was more directional than in denser neuropil of finer cellular structures. Further, we identified that the perisynaptic ECS geometries around respective glutamatergic and GABAergic synapses imposed distinct functional advantages, shedding light on the longstanding conundrum of why glutamatergic and GABAergic synapses are so conspicuously morphologically different.

**Conclusions:**

Our modelling broadly identifies ECS structure as a direct modulator of extrasynaptic signaling that can operate in parallel to conventional regulation mechanisms. This ultimately provides a metabolic and computational advantage to the brain.

**Supplementary Information:**

The online version contains supplementary material available at 10.1186/s12987-026-00797-3.

## Introduction

The brain extracellular space (ECS) is a geometrically complex reticular continuum filled with interstitial fluid and stationary extracellular matrix (ECM) components [14, 23]. It facilitates diffusional transport of signaling molecules, ions, and metabolites to and from cells [37]. Yet, despite its fundamental importance, it remains unknown if the ECS geometric structure modulates diffusion across any scale in a manner that supports specific physiological functions [25]. This is because existing knowledge is provided mostly through experiments on the scales of seconds and hundreds of microns [For reviews, e.g. Syková and Nicholson [38], Tønnesen [43], though this is far from the range of individual point-source transmitter release events that are the basis of neuronal signaling and which unfold on millisecond and nano- to micrometer scales [20]. Diffusion experiments on coarser scales inevitably volume-average data across space and time, and any information about biological variation around the average remains inaccessible. There is a particular lack of knowledge on how neurotransmitters may undergo local anisotropic diffusion in the ECS before finding their target, and accordingly whether ECS geometry serves a role in signal modulation. This question becomes even more pertinent considering that the diffusional properties of the ECS change with aging, trauma, and pathology [38], which are conditions associated with changes in signaling and neuronal plasticity in general.

The two archetypical ionotropic neurotransmitters of the mammalian brain are excitatory glutamate and inhibitory gamma-aminobutyric acid (GABA). Glutamatergic signaling through independently tunable AMPA receptor-rich glutamatergic synapses operates with typical deactivation time constants around three milliseconds and is considered at the core of neuronal wiring of cognition and learning [15]. Outside the synapse, glutamate still serves as a transmitter, though extra-synaptic glutamatergic receptors serve a less clear-cut role and is reportedly not a general modulator of excitability [26]. GABA-A receptors activate synaptic conductances with deactivation time constants of tens of milliseconds to cause fast, phasic inhibition [5]. Additionally, GABA spills over from the synapse and diffuses to peri- and extra-synaptic areas where it gives rise to a tonic inhibitory current mediated also primarily by GABA-A receptor subtypes [1, 5, 29]. Extrasynaptic tonic GABAergic inhibition operate across cell types and brain regions where it amounts to 30–50% of total inhibition and ubiquitously sets baseline excitability [3, 4, 35]. This identifies the complementary two main synaptic transmitter systems as not merely functionally opposite, but also differently implemented and regulated in terms of time scales and extra-synaptic effects.

In addition to their functional differences, the structural geometries of GABA and glutamatergic synapses differ systematically. While the pre-synaptic sides appear largely similar as axonal boutons, the post-synaptic sides differ and the differences are conserved across species and cell types, which suggests a physiological advantage [10, 33]. On most neurons, including hippocampal CA1 pyramidal cells, glutamatergic post-synapses are located on dendritic spines protruding to offset the synapse from the surrounding dendritic membrane. It has been reported that this morphologically compartmentalizes intracellular signal propagation to and from individual synapses [36, 41]. By contrast, GABAergic post-synapses predominantly form directly on the soma or dendritic trunk and are level with the surrounding membrane [18, 21]. GABAergic synapses are therefore not morphologically compartmentalized, and it remains an enigma whether there is a biological advantage of membrane level somatodendritic GABAergic post-synapses and why the two main transmitter systems are so consistently morphologically different.

We hypothesized that the respective geometries of excitatory glutamatergic and inhibitory GABAergic synapses will differently modulate perisynaptic diffusion and spill-over to neighboring synapses and extrasynaptic receptors. Specifically, we propose that diffusional clearance from spines is facilitated so that synaptic crosstalk is minimized, whereas somatodendritic synapse geometry by design enhance lateral diffusion to nearby extrasynaptic receptors to enhance tonic inhibition. To test this, we used fluorescence super-resolution shadow imaging (SUSHI) to image the ECS and neuropil geometry in live mouse brain slices [42]. We first analyzed the ECS volume fraction, α, and the sizes of the imaged ECS geometries as spatial frequencies, and then built a computational diffusion model to simulate diffusion in SUSHI images and test how these parameters could impact perisynaptic diffusion. The model hinges on Fick’s law stating that diffusional flux through a compartment scales with its cross-sectional area. We extrapolate this to SUSHI image pixels and determine to what extent these are a diffusionally accessible part of the imaged ECS, or whether they are instead occupied by cellular structure and inaccessible. We further performed Monte Carlo simulations of single molecules diffusion in SUSHI images using a similar strategy. This allows analyses of diffusion from simulated glutamatergic and GABAergic synapses into their perisynaptic ECS. Lastly, we simulate different frequencies of synaptic GABA activity through somatodendritic synapses to learn if regular point-source release could establish steady state micro-gradients of GABA along the cell membrane to augment local extrasynaptic tonic inhibition.

The combination of super-resolution imaging in live brain slices and computational diffusion modelling provides a unique opportunity to investigate perisynaptic diffusion following individual release events at nano- and micrometer scales, beyond the reach of volume-averaging approaches. Our results points to the ECS as a signal regulator with transmitter system-specific functional roles for respective glutamatergic and GABAergic synapses.

## Methods

### Organotypic mouse brain slices

We prepared organotypic hippocampal slices from postnatal day 5–7 C57BL/6J mice (RRID: MGI:5658686), as previously reported in detail [42]. In brief, hippocampi were sliced at 250 μm on a vibratome (VF-300-0Z, Precisionary) and embedded in a plasma-thrombin clot on a 12 × 24 mm glass coverslip (Thickness #1) before culturing in a roller-drum incubator at 10 RPM in tubes with 0.75 ml medium consisting of 50% minimal essential medium (MEM), 25% horse serum and 25% Hank’s buffered saline solution (HBSS) supplemented with 12 mM glucose and penicillin/streptomycin (all from Gibco) [7]. Cultures were imaged after 2–4 weeks of culturing (protocols.io).

### Super-resolution STED and SUSHI imaging in live brain tissue

We used a custom-built microscope based on an inverted body (DMi8, Leica) with an 93x and 1.3 numerical aperture (NA) glycerol immersion objective with a motorized aberration correction collar (Leica). The STED beam was derived from a 592 nm 500 ps pulsed laser (Katana 06, NKT Photonics), and the excitation beam from a 485 nm 90 ps pulsed laser (Quixx, Omikron). These were brought into temporal phase overlay using the built-in pulse-sync and pulse-delay module of the 485 nm laser. Resolution enhancement was achieved by splitting the STED beam and sending the two parts through a 2π vortex phase plate (doughnut point-spread function [PSF]) and a 1π helical phase plate (bottle beam PSF; both plates from Vortex Photonics) before recombining them to achieve x, y,z resolution enhancement of the emission PSF [42]. We primarily imaged with majority power in the bottle beam PSF for z-resolution enhancement and using STED power of around 15 mW in the objective back aperture, adjusting the settings to the given imaging session. The 15 mW STED power typically provide a volume resolution around 125 × 125 × 300 nm^3^ on our setup, and we would tune power up or down during individual imaging sessions. Pixel and voxel size was in the range of 20 to 100 nm laterally and 100 to 500 axially, with pixel dwell-times within 10 to 50 µs. The laser beams were scanned in x, y by means of a Yanus IV galvanometric scan head (ThermoFisher), and the back-scanned fluorescence was detected using an avalanche photo-diode detector (APD, Laser Photonics) coupled to a 50 μm multimode fiber acting as an optical pinhole for confocal detection (Thorlabs). Z-axis scanning was performed using a piezo-focuser (PIFOC, Physik Instrumente). The microscope was controlled through an FPGA-based data acquisition card using designated Imspector software (Abberior Instruments).

For imaging on our inverted STED microscope, a glass coverslip with a slice culture was glued onto a custom-made polycarbonate imaging chamber using UV glue, so that the glass coverslip effectively became the chamber bottom to allow single-interface imaging from beneath. The slice imaging chamber was perfused with artificial cerebrospinal fluid (aCSF) containing 126 mM NaCl, 2.5 mM KCl, 2.5 mM CaCl_2_, 1.3 mM MgCl_2_, 20 mM glucose, and 27mM HEPES, with pH adjusted to 7.3 at 32–35 °C. To visualize the ECS we used the protocol described in [42]. In brief, we added 20–30 µM calcein to the perfusion solution, which distributes homogeneously in the ECS without entering cells and allows super-resolution STED imaging of the neuropil. For 2-color SUSHI images of dendritic spines in their neuropil context we used previously published images from [42] with permission.

**Fig. 1:**
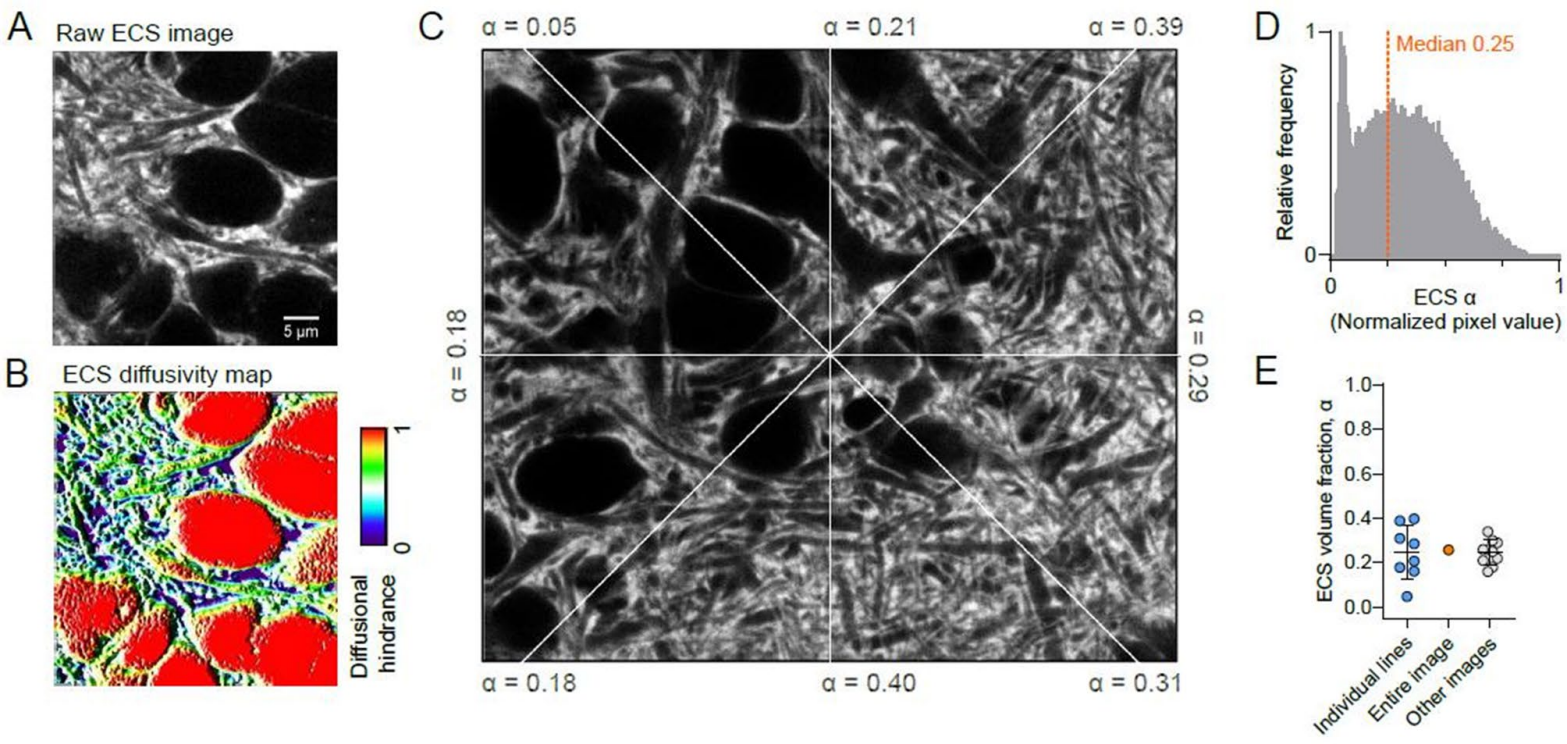
Diffusion model and local geometric and diffusional properties of ECS. (**A**) Our diffusion model utilizes the ECS structural information of SUSHI images to effectively build a diffusional accessibility map (**B**). (**C**) Line profile analysis of radial lines from the image center returns widely varying α values between 0.05 and 0.4. (**D**) Intensity-normalized pixel histogram of image in (**C**), where pixel values directly correspond to α. The median of 0.25 is depicted by dotted line. (**E**) Calculated α for line profiles and image in (**C**) as well as for other images (n = 10 images, 8 slices, 6 animals; Mean and SD)

Images were minimally processed by applying a 1-pixel median filtering to remove single pixel noise arising from photo-detector dark counts (Fig. 1A). The image fluorescence signal histogram was normalized by adjusting for background, $\:{F}_{bckg}$, by imaging inside somas devoid of fluorophores, and for the maximum observed value, $\:{F}_{max}$, obtained by imaging a fluorophore filled ECS volume. Each pixel in a given intensity normalized 8-bit image would accordingly have a value

1 $$\:{F}_{pix}=\:\frac{{F}_{raw}-{F}_{bckg}}{{F}_{max}-{F}_{bckg}}\:\cdot \:255$$

Where *F*_*raw*_ is the unadjusted raw pixel value.

### Modelling ECS diffusion fluxes on nanometer and microsecond scales

Diffusional fluxes in a homogeneous medium scale with the diffusion coefficient, *D*, of the observed particle, and diffusion is spatially confined by the delineating compartment borders. To model ECS diffusion we assumed an ECS filled with homogeneous interstitial fluid resembling saline and delimited structurally by the ECS geometry. In this scenario diffusion is free in pixels filled with pure 100% ECS, whereas diffusion cannot take place in pixels representing pure cellular structure (0% ECS). Diffusion is partially obstructed in pixels partly occupied by cellular structure (0% < ECS < 100%) (Fig. 1B). The ECS geometry-weighted effective diffusion coefficient in a given pixel, $\:{D}_{pix}$, will thus be a function of the pixel ECS volume fraction, $\:{\alpha\:}_{pix}$ that can be easily read out as the relative pixel intensity *F*_*pix*_ / 255.

We assume a linear mapping from the calculation $\:\alpha\:$ to $\:{D}_{pix}$:

2 $$\:{D}_{pix}\left(x,y,z\right)=\:{\alpha\:}_{pix}\cdot\:{D}_{free}$$

Our model was built in MATLAB (Natick, MA) based on a diffusion equation for mass transport in porous brain tissue [22, 24] assuming no advective interstitial fluid bulk flow.

3 $$\:\frac{\partial\:C}{\partial\:t}=\nabla\:\cdot\:{D}_{pix}\left(\nabla\:C\right)+S-\kappa\:C$$

Where $\:C$ is the concentration, $\:S$ is the source magnitude, defined as the amount of substance delivered per unit time and $\:\kappa\:$ is the non-specific clearance coefficient [2]. We used this coefficient to consider diffusion of molecules in the z-axis *(*$\:\kappa\:z$) and thus build a simplified 3D model to model diffusion in 2D images.

In point source release simulations $\:S$ is the number of particles per volume unit at time zero:

4 $$\:S=\frac{n}{{N}_{A}\cdot\:\left(\varDelta\:x\cdot\:\varDelta\:y\cdot\:\varDelta\:z\right)}$$

Where *n* is the number of particles, *N*_*A*_ is Avogadro’s number and ∆x, ∆y, ∆z are the image pixel or voxel sizes. For all our simulations we assumed leaky boundary conditions whereby molecules can escape the image frame at border pixels to mimic a surrounding infinite field of ECS. We applied an image x, y plane standardized border escape factor, *ef*, of 0.9 to simulate 10% of molecules in a given border pixel escaping out of the frame for a given time step, so that for a border pixel located at (x=1,y,z) with a neighboring pixel at (x=2,y,z) the concentration is given by $\:{C}_{t}^{1,y,z}=ef\cdot\:{C}_{t-\varDelta\:t}^{2,y,z}$. The *ef* is based on iterative simulations in a homogeneous field and evaluation of diffusion cloud circularity . For a visual example of border escape, we refer to the simulations in homogenized ECS images further below. We solved the diffusion equation numerically using the Forward Euler Method with time steps on the scale of 1 µs and spatial gradients defined by the resulting pixel molecular concentrations and pixel sizes. Pixel sizes were between 20 and 120 nm, set to be smaller than half of the estimated optical resolution of a given acquisition. Pixel sizes are embedded in the images available through Zenodo.    

Simulations were performed in 2D and 3D as indicated. Further, to reduce the computational cost of 3D simulations and increase the applicability of our model we developed a simplified 3D diffusion model approach, *3D*_*smpl*_ to model diffusion in a given x, y image frame without specific knowledge about the above and below image z-planes and still allowing escape from the plane. To account for 3D diffusional escape out of a given SUSHI x, y plane we introduced a pixel clearance coefficient, $\:{\kappa\:}_{pixel}=\:{\upalpha\:}\mathrm{*}\kappa\:\left(t\right)$, where clearance is higher at pixels with higher ECS volume fraction and it decreases exponentially with release time with a minimum clearance constant, $\:{\kappa\:}_{min}$.

5 $$\:\kappa\:\left(t\right)=15000\cdot\:dt\cdot\:{e}^{\left(-6000\cdot\:t\right)}+{\kappa\:}_{min}$$

Where, $\:{\kappa\:}_{min}=75\cdot\:{10}^{6}\cdot\:{dt}^{2}$. The constants and $\:{\kappa\:}_{min}$ scaling factor were iteratively determined to replicate actual 3D imaging-based modelling of peak concentration, time to peak, and exponential decay time constants across experiments (Suppl. Figure 1).

For simplicity we refer to our extracellular space diffusion flux model as *DifFlux*.

### Monte Carlo diffusion modeling in live ECS geometries

The diffusion of single particles is defined by their probability, $\:p$, to randomly displace from one location to another at every $\:{\Delta\:}t$. Like in the *DifFlux* approach, for Monte Carlo simulations we assumed a linear mapping of $\:p$ as a function of $\:\alpha\:\left(x,y,z\right)$ in a given SUSHI image, again exploiting the ECS structural information available in each pixel. At every step a random number is used to select an axis of movement with equal probability, $\:{p}_{dir}=\frac{1}{2}\:\rm or\:\frac{1}{3}$, depending on the number of dimensions, 2D or 3D. Another random number chooses the direction along the axis with probability $\:{p}_{i}=\frac{1}{2}$. We then use a homogeneously distributed random number, $\:r$, to simulate bouncing from an obstacle in the new selected location, $\:{p}_{b}=(1-\:\alpha\:\left(x,y,z\right))$. If, $\:r<{p}_{b}$ then the particle stays in the original voxel, as described in our previous work [32]. The Monte Carlo model assumes toroidal boundary conditions and non-interacting particles.

### Analysis and statistics

All modelling and analyses were performed in raw images subjected to an evenly applied 1-pixel median filter to remove single pixel detector noise. Figure images are not further processed. The ECS *α* for an image frame or line profile was calculated as the median pixel value divided by 255 (corresponding to *α* = 1) in the intensity-normalized images. We tested data for normality and applied parametric or non-parametric testing and descriptive statistics as specified for results throughout. We considered probabilities less than 0.05 significant.

## Results

### The ECS volume fraction is highly heterogenous and imparts anomalous diffusion

The normalized fluorescence intensity value of each pixel in each image corresponds to the ECS volume fraction *α*. We found that *α* varied considerably within images as well as between images and brain slices. Radial lines from the center of the SUSHI image depicted in (Fig. 1C) reported ECS α values varying 8-fold between 5% and 40% with a mean and SD of 0.25 ± 0.12 (*n* = 8 lines), in excellent agreement with the α = 0.26 based on integrating all pixels of the image (Fig. 1D). The mean α value again fits well with the average across other *stratum radiatum* images from different slices where *α* = 0.25 ± 0.06 (*n* = 10 slices, 6 animals) (Fig. 1E). The observation of widely different α values of line profiles emerging from a common center within a given image suggests that diffusion will also depend on the considered diffusion direction from a source point, so that a given tissue tortuosity is valid only for a given diffusion direction. While α varied widely within and between tissue areas, the average was robust around 25%, in agreement with literature values [17, 19, 44].

**Fig. 2:**
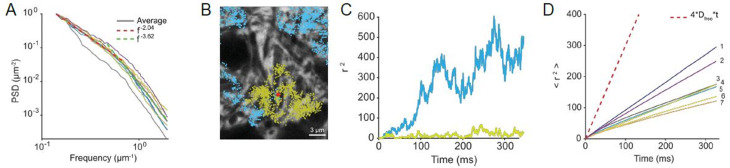
Monte Carlo simulations of ECS diffusion. (**A**) Fourier based radially averaged power spectral density (PSD) analysis of ECS geometry from seven SUSHI image stacks shows a continuous distribution with apparent adherence to power law. The average PSD across all 7 stacks shown in black, the fitting exponent β = 2.04 (95% CI: 2.02- 2.06) (red dotted line) at low frequencies and β = 3.62 (95% CI: 3.58- 3.66) at high frequencies (green dotted line). (**B**) Two examples of Monte Carlo simulation of particle trajectories after release at a common source point (red), illustrating different resulting trajectories (blue and green) and (**C**) mean squared displacement (r^2^) values. (**D**) Average mean squared displacement (<r^2^>) values for middle plane of each of seven stacks stack illustrating anomalous diffusion relative to free diffusion (dotted line). A total of 50 random source point release events were simulated per image plane. A power law fit to the average <r^2^> plot yielded an overall exponent, γ, of 0.88 ± 0.02 SEM, n = 7.

We further quantified the structural heterogeneity of the ECS by 2D radial Fourier analysis in live tissue SUSHI z-stacks (*n* = 113 planes, 7 image stacks/slices/animals) [30]. The radially averaged power spectral density (PSD) shows a smooth transition from low to high spatial frequencies indicating a continuum of ECS geometries, again corroborating earlier results of lognormal distributions [8, 42] (Fig. 2A). Our analysis focused on spatial frequencies ranging from $\:{0.13\:\mu\:m}^{-1}$, corresponding to large-scale features, to $\:{2\:\mu\:m}^{-1}$, which reflect image noise as determined by analyzing dark regions in the images. Given the high spectral similarity within each stack, we averaged PSDs per stack and then computed a grand average across all stacks. We identified two regions in the averaged PSD that can be approximated by a power law, $\:PSD\sim1/{f}^{\beta\:}$. The lower spatial frequencies, $\:0.13-0.5\:{\mu\:m}^{-1}$, dominated by neuronal somas and large dendrites, with a fitted exponent of $\:\beta\:=2.04\:\:(95\%\:CI:\:2.02-2.06)$. This corresponds to Brownian noise, indicative of large-scale, smooth, and correlated ECS structure similar to natural images [6, 28]. Such structure suggests long-range correlations akin fractals and disordered media [11, 12]. The high frequencies ($\:0.5-2\:{\mu\:m}^{-1}$) were dominated by finer ECS structures, with a fitted exponent of $\:\beta\:=3.62\:(95\%\:CI:\:3.58-\:3.66)$. This value of $\:\beta\:$, sometimes called super-Brown or colored noise, reflects suppression of fine-scale detail, consistent with smoother extracellular boundaries or blurred fine structure, rather than sharp discontinuities. This may indicate a breakdown of fractal behavior at small scales potentially due to limitations in optical resolution which could smooth the images at small scales, masking actual fractal behavior. The PSD showed consistency between slices, suggesting a conserved ECS geometry within the CA1 region (Fig. 2A). Our combined α and Fourier analyses show that the ECS geometry is highly variable yet conserved with comparable average α and geometry within the CA1 *stratum radiatum*.

To explore how ECS structural heterogeneity influences molecular diffusion we applied 2D Monte Carlo simulations of diffusion of a small molecule (*D*_*free*_ = 0.5 µm^2^/ms). First, we observed that *DifFlux* and Monte Carlo simulations yielded comparable results when applied to simulate diffusion using similar parameters (Suppl. Figure 1). Using Monte Carlo simulations, we tracked the movement of 1,000 glutamate sized particles (*D*_*Glu*_ = 0.75 µm^2^/ms) released from 50 randomly selected source points over 300 ms using toroidal boundary conditions. Plotting the individual particle trajectories emerging from a given release site showed large variability in diffusion patterns, (Fig. 2B, C). We calculated the mean squared displacement (MSD, $\:<{r}^{2}>$) for each source point to determine their average behavior. For each image frame, we averaged the MSD curves from 50 simulations. This showed slowed-down non-linear diffusion characteristic of anomalous diffusion (Fig. 2D) (*n* = 7 frames/slices/animals). Indeed, a power law fit, < r^2^ > ~ t^γ^ , where γ is the diffusion exponent, described the average MSD well with an overall value of $\:\gamma\:$ of $\:0.88\:\pm\:\:0.02\:SEM$ (*n* = 7 images). This deviation from normal diffusion ($\:\gamma=1$), was statistically significant in all cases (t-test and Wilcoxon test, *p* < 0.0001). Taken together, our Monte Carlo simulations corroborate that the *stratum radiatum* neuropil is geometrically heterogeneous, characterized by power-law geometric structure, and is associated with pronounced anomalous diffusion. Brain parenchyma anomalous sub-diffusion has been reported on volume-averaged scales though it has not been previously related to actual ECS geometries [45].

### Tensor mapping identifies diffusion directionality based on ECS geometry

**Fig. 3:**
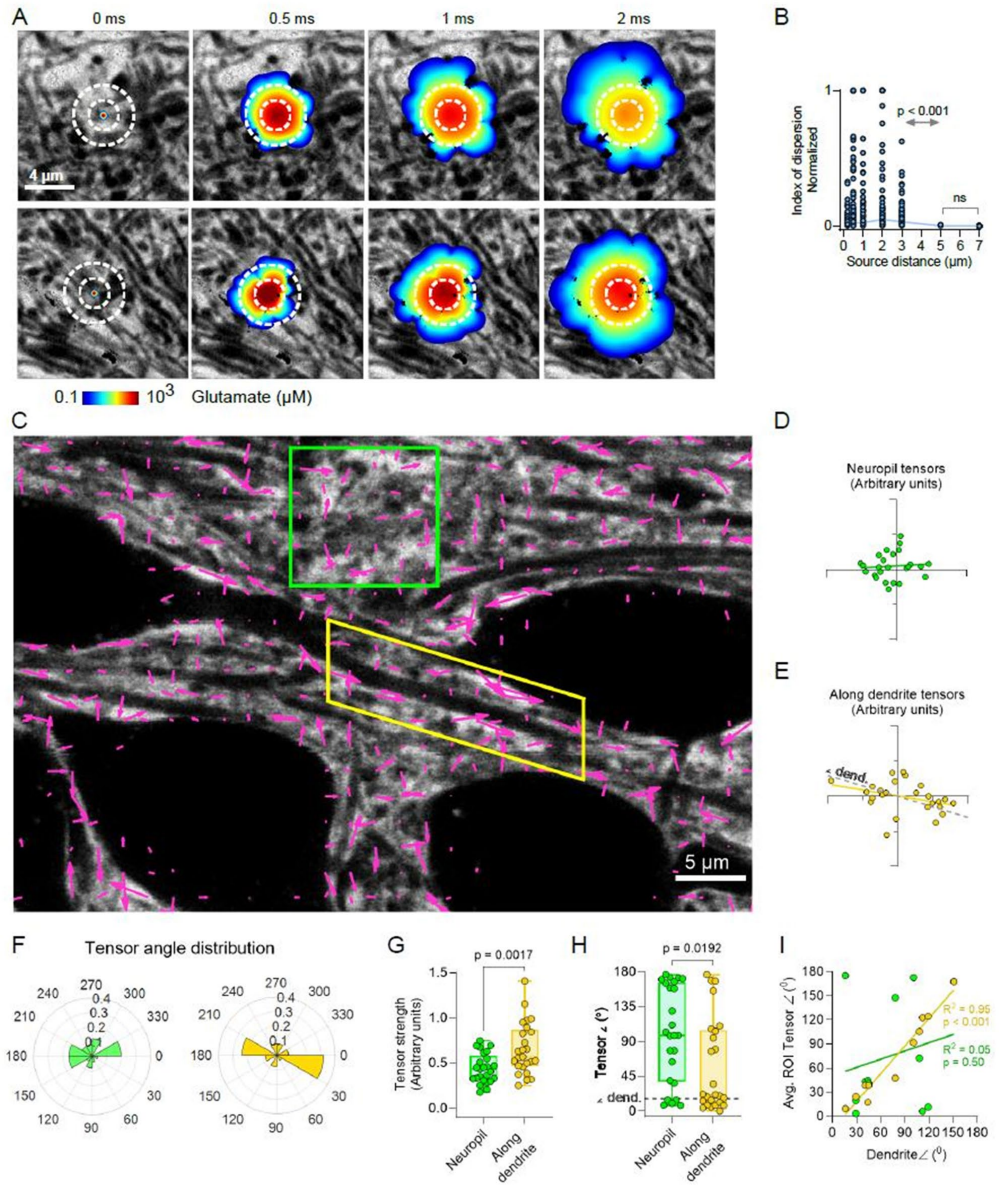
Local diffusion directionality is determined by ECS geometry. (**A**) Two examples of DifFlux simulations of glutamate release in dense neuropil. Dotted circles shown for reference across time lapse. (**B**) Normalized index of dispersion of resulting glutamate concentrations at indicated source distances for the two examples in (**A**) and other slices illustrating that the variation is highest below 5 µm from the source point (n = 5 slices/animals, Kruskal-Wallis test). This confirms anisotropic diffusion at micron and submicron scales that diminishes at larger distances. (**C**) ECS diffusion tensor map showing local directionality (angle, and integrated tensor strength with goodness of fit (as length). (**D**) Tensors in dense neuropil (green box in **C**) appear scattered and random, (**E**) while along dendrites diffusion tensors appear to follow the direction of the extending dendrite. The dotted line indicates the dendrite extension angle. (**F**) Binned (30 degrees) directionality angle distribution of tensors show that in dense neuropil there is less clustering around specific angles than along dendrites where the vast majority of tensors fall in the bins0°-30° and 180°-210° that encompass the 16°/196° dendrite extension angle. (**G**) Comparing tensor strengths reveals that these are stronger along dendrites 0.66 ± 0.29 μm, mean ± SD) than in dense ECS neuropil (0.44 ± 0.16 μm, mean ± SD; Unpaired t-test, p = 0.0017). (**H**) Similarly, the 0-180° orientation of tensors significantly differed between dense ECS where tensors were distributed throughout the interval, and along dendrite where again the majority of tensors were clustered close to the 16° angle of the dendrite itself (dotted line). (**I**) analysis across experiments found that the angle of tensors along a dendrite would strongly correlate with the angle of the dendrite (R^2^ = 0.95, n = 12 frames, 10 slices, 6 animals), while in the same images tensors in dense neuropil would have a seemingly random distribution with respect to the dendrite (R^2^ = 0.05)

To further explore how ECS geometry could shape local molecular diffusion in the interstitial space we used *DifFlux* to simulate release of glutamate molecules (*D*_*Glu*_ = 0.75 µm^2^/ms) from individual point sources in SUSHI images of dense CA1 neuropil (Fig. 3A). We found that the normalized index of dispersion, corresponding to the variance, of resulting concentrations at given distances around the release site would be median 0.007 at 0.2 μm and reaching maximum of 0.047 at 2 μm then come down to become insignificant beyond three microns compared to all lesser distances (*p* < 0.001, *n* = 6 images, 4 slices/animals, Kruskal Wallis test) (Fig. 3B). This shows that there is pronounced anisotropic diffusion and high variability at single micron scales immediately after release in *stratum radiatum* neuropil, though at larger scales the variation is canceled out. Functionally, this would mean that the ECS can have a stronger modulatory effect on diffusional processes on scales below a few microns.

We next asked if point-source diffusion anisotropy was generally random or could translate into diffusional directionality across tissue areas, and modelled release of glutamate from 775 point-sources distributed in a regular grid pattern across an image. For each resulting diffusion point cloud we identified the furthest-from-source point and applied linear regression to identify this as main direction measured 100 µs after release (Fig. 3C). The direction of each point was assigned a vector with a corresponding direction (angle) and magnitude (diffusion distance). We further incorporated the observed bias of the anisotropy by scaling the vector magnitude by a directionality coefficient to obtain a corresponding diffusion tensor. The directionality coefficient was determined by first calculating the distance to the source of all the points that describe the diffusion cloud perimeter for a given source point. Then we plotted the cumulative distribution of that distance for each cloud and estimated the area under the curve, with the normalized area under the curve corresponding to the directionality coefficient. Here, if the diffusion pattern goes toward isotropic with a circular spread the tensor magnitude goes toward zero and the tensor is short. Conversely, the stronger the anisotropy, the longer the tensor becomes.

Applying this strategy in a SUSHI image that includes both dendrites and dense neuropil, we found that ECS diffusion tensors appeared with low directionality in dense neuropil (Fig. 3D), while along dendrites ECS diffusion tensors were stronger and following the direction of the dendrite (Fig. 3E). Accordingly, the majority of tensors were found in the direction bins 0°-30° and 180°-210° corresponding to the 16° (and accordingly 196°) angle of the dendrite in the field of view (Fig. 3F). Comparing the respective neuropil and peri-dendritic tensor strengths (speed weighed by goodness of fit), neuropil tensor arbitrary values were 0.44 ± 1.6 (mean with SD, *n* = 25 tensors), while they were 0.66 ± 2.9 (*n* = 25 tensors) along the dendrite (*p* = 0.002, unpaired t-test) (Fig. 3G). Tensor angles were also different between neuropil and dendritic ECS, with the neuropil median of 98° (interquartile range [IQR] 38°-166°) close to random (average 90°), whereas the dendritic ECS tensor median angle was 21° (IQR 9.4°-105°), close to the 16° angle of the dendrite (*p* = 0.192 Mann Whitney U test) (Fig. 3H). This held true across experiments, where the dendritic angle would account for 95% of the variation in ECS tensor angles along dendrites and be significantly non-zero (Linear regression, *R*^*2*^ = 0.95, slope 1.15, non-zero *p* < 0.0001, *n* = 12 frames, 10 slices, 6 animals), while in dense neuropil of the same images the corresponding *R*^*2*^ was 0.05 and the 0.34 slope not different from zero *p* = 0.50 (Fig. 3I). These results suggest that the structural geometry of the ECS shapes diffusion and that not only directionality but also diffusion speed is modulated along membranous structures such as dendrites.

### Glutamatergic synapse crosstalk from diffusional synaptic spillover

**Fig. 4:**
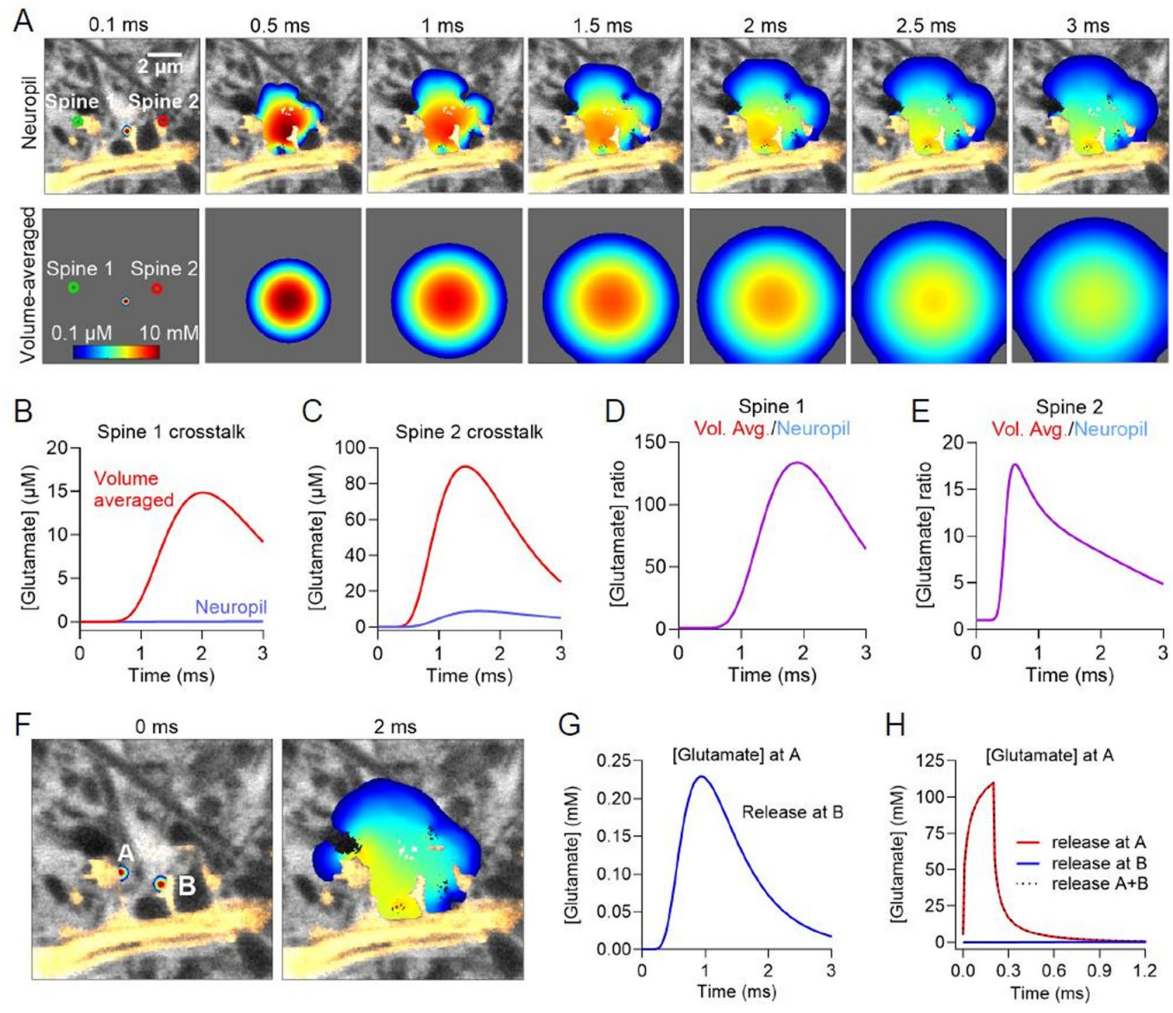
Dendritic spine geometry facilitates extrasynaptic diffusional clearance. (**A**) Simulation of synaptic glutamate release at a dendritic spine with two immediate neighboring spines 1 and 2, and the corresponding frame volume-averaged to eliminate any structural information. (**B**) Peak glutamate reaching neighboring Spine 1 through synaptic crosstalk is 14.8 μM in the case of volume averaging, while in the SUSHI image with neuropil context the maximum reached concentration is merely around 0.04 μM. (**C**) Similarly for neighboring Spine 2, in the SUSHI image case the maximum crosstalk reaches 8.8 μM while in the volume-averaged case it is 89.5 μM. The ratios of crosstalk in the volume averaged and neuropil context amounted to 134 for Spine 1(**D**) and 18.0 for Spine 2(**E**), suggesting that in these two cases the neuropil greatly reduces crosstalk compared to the volume averaged scenario. (**F**) Moving the neighbor Spine A synapse even closer to the source spine B, we analyzed simulated glutamate concentrations at A. (**G**) Crosstalk now reached 0.22 mM. (**H**) Still this crosstalk appeared negligible compared to the concentrations reached after direct glutamate signaling onto A where concentrations surpassed 100 mM

After observing local diffusional directionality imposed by ECS geometry, we tested if perisynaptic ECS geometry modulate extracellular synaptic crosstalk of glutamatergic synapses on spines. Single vesicular release of glutamate was simulated onto a post-synapse on a dendritic spine and the resulting concentrations at two neighboring spine synapses was analyzed (Fig. 4A). Both neighbors were roughly 2 μm from the central source synapse and the spread of glutamate was simulated over 3 ms following release. We compared this SUSHI scenario to the same simulation in a volume averaged frame where *α* is similar to the SUSHI frame (measured *α* = 0.27) but there is no structure (Fig. 4A).

We observed that having knowledge on the ECS geometric context would dramatically affect the resulting crosstalk, and that diffusional clearance appeared faster in the neuropil context than predicted from volume-averaged data. Notably, neighboring Spine 1 would see more than 100-fold higher glutamate concentrations in the volume averaged case, while neighboring Spine 2 would see more than 10-fold higher concentrations in the volume averaged case (Fig. 4B-E). This suggests that for the given case the perisynaptic spine ECS geometry facilitates synaptic clearance and minimizes extracellular crosstalk beyond the levels predicted based on averaged data. We wondered how this level of crosstalk would compare to the glutamate concentration a synapse sees upon direct signaling, and to our surprise the added effect of crosstalk for a 1 μm distanced spine pair turned out to be practically indiscernible from direct signaling alone, suggesting the crosstalk effect is in this case and context negligible (Fig. 4F-H). While this is simply one example, it exemplifies how glutamatergic synaptic crosstalk can be shaped by ECS geometry to be more than 100-fold less than predicted from volume averaged data, which would reduce signaling noise and enhance fast independent signaling at glutamatergic synapses.

### ECS geometry facilitates extrasynaptic tonic inhibition from diffusional GABA synapse spillover

**Fig. 5:**
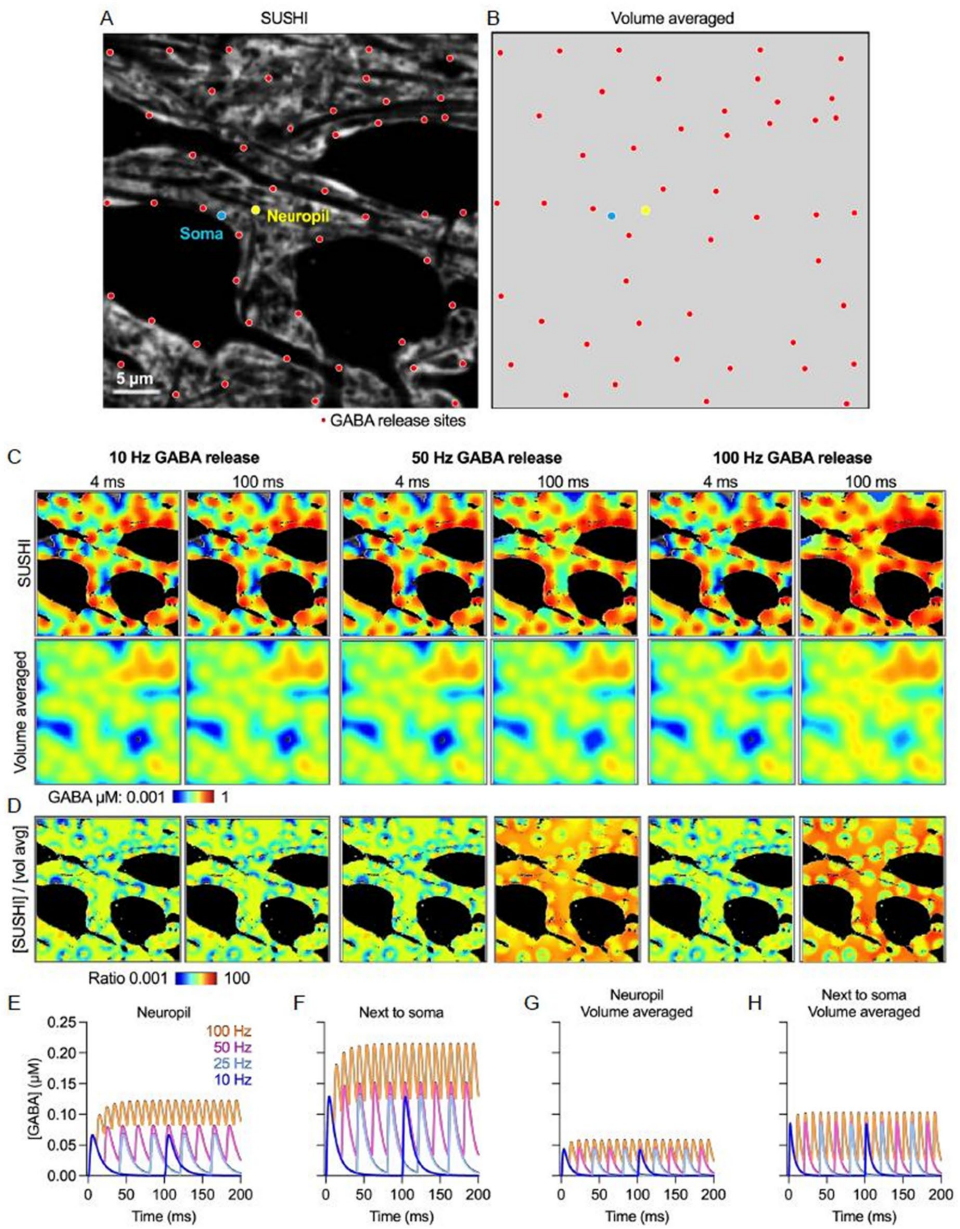
ECS geometry shapes GABA spillover to enhance extrasynaptic inhibition. (**A**, **B**) SUSHI image and the equivalent volume-averaged image. The images show GABA release sites distribute 5 μm away from each other (red dots), a measuring point next to the soma (blue) and in the neuropil (yellow). (**C**) Representative simulations of GABA release at 10 Hz, 25Hz, 50 Hz and 100 Hz. Images show the GABA distribution after 4 and 100 ms for the SUSHI and the volume-averaged image. (**D**) Ratio between the observed concentrations in the SUSHI and volume-averaged image. The ratio is displayed for 10Hz, 50 Hz and 100 Hz GABA release frequencies, at 4ms and 100ms into the stimulation sequence. (**E**, **F**) Extracellular GABA concentration measured over time in neuropil and next to the soma in the SUSHI image, and correspondingly in the volume-average image (**G**, **H**)

We turned from protruding glutamatergic spine synapses to membrane-level somatodendritic GABAergic synapses to learn how ECS geometry here influences perisynaptic diffusion. Vesicular GABA release was simulated in a SUSHI image at 47 distributed somatodendritic and dense neuropil release points separated by around 5 μm from each other and using *D*_*GABA*_ = 1.1 × 10^− 9^ m^2^/s [16, 27] (Fig. 5A). The chosen synapse density is in the range of around 0.1 per µm reported for CA1 pyramidal cells [39]. At each site 10,000 molecules were released at 10, 25, 50 and 100 Hz that is within the published firing ranges of GABAergic interneurons [13]. Again, the results were compared to a corresponding homogenized image that has the same average α but discards ECS structure (Fig. 5B).

We observed in the SUSHI image simulations that at increasing release frequencies the extracellular GABA concentration plateau grows to reach a higher steady-state (Fig. 5C-F). This increase was more pronounced along somatic cell membranes of the receiving neuron, indicating that the large smooth ECS geometry at the soma membrane surface facilitates lateral diffusion from somatodendritic synapses to nearby extrasynaptic receptors, corroborating our tensor mapping above (Fig. 5E-F). Measuring the corresponding concentrations at the same sites in the volume averaged image without ECS structure, this build-up of extracellular GABA was several times less pronounced, highlighting the applicability of *DifFlux* for nano- and microscale simulations (Fig. 5G-H).

Our results suggest that somatodendritic synapse geometry facilitates lateral diffusion along the membrane and thus spillover to perisynaptic receptors and extrasynaptic GABAergic inhibition. Interestingly, it suggests that for regular release frequencies steady state GABA concentration gradients can build up and extend from the soma surface and into the neuropil, meaning that extrasynaptic receptors on the cell membrane see a higher concentration of GABA than predicted from considering average interstitial GABA concentrations. Again, the steepness and stability of such steady state gradients will be highly context dependent.

## Discussion

Having built a diffusion model and gained confidence in its applicability to reveal diffusion gradients in SUSHI images of live brain tissue ECS, we applied *DifFlux* alongside Monte Carlo modelling to simulate point-source release of transmitters in the neuropil. We observed anomalous sub-diffusion compared to free diffusion, as also observed by others [9]. We went on to find pronounced anisotropic diffusion on the micron scale, which would cancel out on larger scales that are usually captured by volume-averaging techniques.

These findings draw into question the soundness of applying volume-averaged diffusion models to understand ECS diffusion on single-micron scales where volume-transmitters such as GABA likely exert their main effect. We confined our modeling to relatively homogeneous CA1 *stratum radiatum* ECS, and we expect that modelling diffusion across cell layers of brain regions would identify anisotropic diffusion also at larger scales that encompass distinct cell layers, vessels, or ventricular structures.

We further analyzed directionality of diffusion tensors with reference to ECS geometries and observed that dendrites facilitate diffusion along their direction, while dense neuropil imposes no apparent diffusional directionality. Such directionality results will again depend on the exact location of the point-sources observed and the running time of individual simulations. We note that this is not merely a technical aspect, as it will also apply to biological processes, where the degree of diffusion anomaly, anisotropy and directionality will depend on the given diffusion distance and time from a given point-source event. The notion that ECS geometry shapes diffusion is perhaps unsurprising considering diffusion theory, yet any quantitative analyses incorporating true brain tissue geometry has hitherto been lacking.

The observed differences between diffusion along larger membranes and in denser neuropil prompted us to simulate perisynaptic diffusion of transmitters from archetypical glutamatergic and GABAergic synapse that have very different morphologies. Glutamatergic synapses commonly reside on dendritic spines that effectively offset them from the dendritic shaft and position them in denser neuropil, whereas somatodendritic GABAergic synapses are commonly formed on, and flush with, the somatic or dendritic membrane.

Our modelling found that transmitter clearance from glutamatergic synapses is facilitated by spine geometry to minimize extracellular synaptic crosstalk so that clearance is higher than predicted from volume averaged models. This suggests that dendritic spine geometry enhances perisynaptic diffusional clearance and supports the common view of glutamatergic synapses as individual, independent, fast-tunable substrates of memory and learning [31, 40]. Conversely, we found that the somatodendritic location of GABAergic synapses facilitate lateral diffusion to nearby extra-synaptic receptors on the membrane and locally augment tonic GABAergic inhibition for a given combination of GABA molecules and extra-synaptic receptors. Interestingly, this direct signaling modulation by the ECS can manifest independently of presynaptic quantal size, GABA release frequency, transporter activity, post-synaptic receptor numbers and states, which are the variables by which we currently understand extrasynaptic signaling. In other words, the ECS diffusion regulation mechanism can operate independently of the transmitting and receiving cell pair, and will not be revealed by investigating these.

These findings suggest GABA synapse geometry has evolved in a way that facilitates synaptic as well as spill-over derived extrasynaptic signaling.

Our data add a new ECS perspective to seminal work concluding that tonic inhibition of hippocampal dentate gyrus granule cells emerges primarily from somatic post-synapses because it persists when trimming away the dendritic tree [34]. Our results imply that even for homogenously distributed synapses across dendrites and soma, tonic inhibition of the somatic compartment is higher simply because the ECS volume fraction here is lower and diffusion more restricted, so released GABA will yield higher ambient ECS concentrations [42].

Running the *DifFlux* model in parallel to Monte-Carlo simulations applied in a corresponding analogue manner yielded comparable results. Whereas *DifFlux* represents a bulk model that does not consider trajectories of individual molecules, the Monte Carlo approach allows tracking of individual molecules and analysis of MSD. The advantage of *DifFlux* here is that it is much lighter computationally, though the two are highly complementary.

Our modelling has brought new insights that may be tested through future experimental studies. The current highest resolution empirical approach is single molecule tracking of diffusing single-walled carbon nanotubes that yield both geometric and diffusional data, though a key limitation is that single carbon nanotubes do not distribute homogenously and exhaustively in brain parenchyma [8]. This limits what can be learned about the relationship between ECS geometry and point-source diffusion. Another notable technique is 2-photon excitation time-resolved fluorescence anisotropy imaging that also yields simultaneous diffusional and geometric data, though it lacks the resolution to resolve the brain neuropil, and is restricted in the ranges of diffusion it can resolve [46]. Still, it would be interesting to apply this approach based on STED microscopy to effectively reconcile nanoscale resolution imaging with concurrent ECS viscosity data.

Our *DifFlux* and Monte Carlo models based on perfusion labelling and super-resolved fluorescence microscopy of the ECS in brain slices allows nanoscale analysis of molecular diffusion in actual images of live brain parenchyma. Instead of a binarization and thresholding approach, it adopts a largely analogue approach in raw fluorescence images and exploits the full pixel bit depth to harness 3D information about ECS/cellular structure ratios in individual pixels. While higher spatial resolution gives more accurate results, the model does not necessitate resolving ECS geometry as long as a qualified estimate or measurement of background and maximum observable ECS fluorescence can be made. The model thus requires few underlying assumptions, making it robust and applicable across fluorescence microscopy modalities. It simulates diffusion in 3D and can be applied in 3D images as well as 2D images where binarized models fall short because they produce closed maze ECS geometries.

Our modeling approach is purely structural and blind to ECS viscosity effects or binding/uptake of the diffusing molecules, though both parameters can conceivably be incorporated. Indeed, this could be done in the same SUSHI images by retrospectively mapping onto these immunohistochemically mapped matrix constituents, membrane receptors, transporters, or other targets of interest.

We acknowledge that there are further confounders of our model. Firstly, the background fluorescence across images is expectedly not fully homogenous, leading to an error in our estimates of *α* based on *F*_*Pix*_ that will scale with our signal to noise ratio. This error is expectedly low because of the high contrast of the general volume labelling approach. Further, the microscope point-spread function (PSF) is not entirely homogenous, and a given pixel intensity may therefore not scale completely linearly with pixel *α*. Though the resulting error will expectedly be symmetric and not biased toward lower or higher pixel values. The same confounder applies to our image border escape coefficients in the x, y,z planes, where this is not in reality homogeneous even if we assume it. This is especially true for the z-axis where we for 3D_smpl_ based analyses use a predetermined z-axis escape factor, though our analyses suggest that results are comparable to true 3D modelling, which likely reflects that the contribution of molecular “bounce” between z-planes is low compared to the contribution of molecules moving within a given x, y plane. These confounders presumably add noise without biasing our results, and we accept them *as are* to have an as simple as possible model with minimal assumptions.

## Conclusion

In concert, our perisynaptic diffusion modelling suggests that the very different geometries of glutamatergic and GABAergic synapses support separate functions and increase metabolic and computational efficiency in a transmitter system specific manner. This should be thought of in terms of probabilities, rather than as a ubiquitous truth, as outcomes will depend on the specific physiological context, not least signaling activity levels.

Down the line, our model will allow us to further understand how brain ECS diffusional properties change across brain regions, neuronal plasticity, development and ageing, and how inflammation or neurodegenerative disorders associated with extracellular protein aggregation impact not only signaling, but also metabolite clearance *via* the ECS.

Ahead, it will be interesting to extend the *DifFlux* model to accept 4D SUSHI images and look at ECS structural dynamics, and additionally combine the 4D modelling predictions with functional data, such as calcium-imaging or electrophysiology experiments to further explore how ECS geometry may impact brain function across scales. Adding data from immunohistochemistry, fluorescence correlation spectroscopy, polarization microscopy, and more, we can likely improve the model by incorporating non-structural regulators of ECS diffusion, for example viscosity and molecular binding data. Finally, the *DifFlux* model could also be applied to simulate diffusion in other tissue and to simulate intracellular diffusion.

## Supplementary Information

Below is the link to the electronic supplementary material.

Supplementary Material 1

## Data Availability

All simulations were programmed in MATLAB (Natick, MA). The code and analyses are freely available from Github: DifFlux diffusion model (RRID: SCR_027942; 10.5281/zenodo.18750224) and Monte Carlo diffusion with obstacles model (RRID: SCR_027941; https://doi.org/10.5281/zenodo.18834019). All images, results and key resource identifiers are available through the Zenodo repository (https://doi.org/10.5281/zenodo.18750437).

## Abbreviations

APD: Avalanche photo-diode
GABA: Gamma-aminobutyric acid
ECM: Extracellular matrix
ECS: Extracellular space
HBSS: Hank’s buffered saline solution
MEM: Minimal essential medium
MSD: Mean squared displacement
NA: Numerical aperture
PSF: Point-spread function
STED: Stimulated emission depletion
SUSHI: Super-resolution shadow imaging
